# A Digital Twin Strategy Combined with a Monte Carlo Simulation Framework to Predict Outcomes in Patients with Unusual-Site Venous Thrombosis Treated with Direct Oral Anticoagulants Versus Vitamin K Antagonists Using Data from Real-World Populations

**DOI:** 10.3390/clinpract15120237

**Published:** 2025-12-17

**Authors:** Anabel Franco-Moreno, Luis Escobar-Curbelo, Juan Torres-Macho, Nuria Muñoz-Rivas, Cristina Lucía Ancos-Aracil, Ana Martínez de la Casa-Muñoz, Ana Bustamante-Fermosel, Paz Arranz-García, Miguel Ángel Casado-Suela

**Affiliations:** 1 Department of Internal Medicine, Hospital Universitario Infanta Leonor, 28031 Madrid, Spain; juan.torresm@salud.madrid.org (J.T.-M.); nuriamunoz@salud.madrid.org (N.M.-R.); abustafermo3@gmail.com (A.B.-F.); xelele54@gmail.com (M.Á.C.-S.); 2 Venous Thromboembolism Unit, Hospital Universitario Infanta Leonor, 28031 Madrid, Spain; 3 Artificial Intelligence, 11001 Cádiz, Spain; drcurbelo@gmail.com; 4 Department of Medicine, Universidad Complutense de Madrid, 28040 Madrid, Spain; 5 Department of Internal Medicine, Hospital Universitario de Fuenlabrada, 28942 Madrid, Spain; drancos@gmail.com; 6 Venous Thromboembolism Unit, Hospital Universitario de Fuenlabrada, 28942 Madrid, Spain; 7 Hospital Universitario Gregorio Marañón, 28007 Madrid, Spain; amdelacasa@hotmail.com; 8 Management Control Area, Hospital Universitario Infanta Leonor, 28031 Madrid, Spain; paz.arranz@salud.madrid.org

**Keywords:** bleeding, digital twin, direct oral anticoagulants, artificial intelligence in medicine, Monte Carlo methods, precision medicine, recanalization rate, real-world data, thrombosis recurrence, unusual-site venous thrombosis, Vitamin K antagonist

## Abstract

**Background/Objectives:** Unusual-site venous thrombosis (USVT) lacks robust evidence guiding anticoagulant selection between vitamin K antagonists (VKAs) and direct oral anticoagulants (DOACs). This study aimed to evaluate recanalization, recurrence, and major bleeding outcomes in real-world USVT patients and to replicate these findings through a validated digital twin model with Monte Carlo simulation. **Methods:** We conducted a retrospective study of 90 USVT patients (72% VKAs, 28% DOACs). A conditional generative adversarial network was used to generate digital twins matched on age, sex, thrombosis site, and malignancy. Logistic regression was applied to estimate treatment-specific outcome probabilities for recanalization, recurrence, and major bleeding. A nested stochastic simulation framework simulated 500 iterations across clinical scenarios, including increased DOAC use, cancer prevalence, cerebral vein thrombosis proportion, and optimized VKA control. **Results:** The mean age was 67.5 years, and 54.4% were female. 61.1% of splanchnic vein thrombosis, 36.7% of upper extremity deep vein thrombosis, and 2.2% of cerebral vein thrombosis were included. In the real cohort, complete recanalization occurred in 40.0% of patients with DOACs and 36.0% with VKAs. Recurrence was 8.0% with DOACs and 7.7% with VKAs, and major bleeding occurred in 8.0% and 10.8% of cases, respectively. All-cause mortality was 20% in DOAC-treated patients and 60% in those receiving VKAs. Digital Twin-based predictions replicated these results (recanalization 40.3% versus 38.0%; recurrence 10.9% versus 8.6%; bleeding 7.6% versus 9.1%). Simulated scenarios preserved the directionality effect, with the most significant differences observed in high-cerebral vein thrombosis and cancer-enriched patients. **Conclusions:** DOACs showed comparable efficacy and slightly lower bleeding risk than VKAs in USVT. Digital twin and Monte Carlo modeling provided robust, reproducible simulations of treatment effects under varying clinical conditions. Separating empirical and simulation-based findings, the digital twin supported the internal consistency of real-world observations and demonstrated the potential of in silico modeling as a complementary tool in rare thrombotic diseases.

## 1. Introduction

Unusual-site venous thrombosis (USVT) refers to thrombosis occurring in venous territories other than the lower extremities and pulmonary arteries. Atypical sites include the deep veins of the upper extremities, cerebral sinus veins, and splanchnic veins (portal, mesenteric, and splenic veins).

In the absence of contraindications, anticoagulant therapy should be initiated promptly after USVT diagnosis. Direct oral anticoagulants (DOACs) have become the gold standard for treating venous thromboembolism (VTE). DOACs offer several advantages over vitamin K antagonists (VKAs), including predictable pharmacokinetics, fewer drug and food interactions, and rapid onset and offset of action. In addition, DOACs are associated with lower rates of intracranial hemorrhage, which has led to their broader—and sometimes off-label—use [1].

Several randomized controlled trials (RCTs) have evaluated the efficacy and safety of DOACs for treating USVT [2,3,4,5,6,7,8,9] (Supplementary Table S1). Evidence suggests that DOACs are at least as effective and safe as VKAs, showing similar rates of recurrent thrombosis, major bleeding, and recanalization. However, the RCTs are limited in size and have low statistical power, so the results should be interpreted with caution. Current evidence supports DOACs as a reasonable alternative to VKAs in patients with USVT, but larger studies are needed to confirm these findings [10,11,12]. Detailed descriptions of the RCTs are available in the Supplementary Material.

CVT (ORPHA: 329217), non-malignant and non-cirrhotic PVT (ORPHA: 854), isolated mesenteric vein thrombosis (ORPHA: 583861), and isolated splenic vein thrombosis (ORPHA: 583856) are recognized as a rare disease by the Orphanet database. Given the very low incidence of USVT, generating robust evidence through additional RCTs or prospective cohort studies would require prolonged follow-up periods and substantial recruitment efforts.

Given the low incidence of USVT, generating solid evidence through other RCTs or prospective studies would require many years and large-scale recruitment efforts.

The use of digital twin (DT) models represents an innovative and powerful approach to address the methodological limitations of research in rare diseases or low prevalence. A DT is a computational replica of a real-world patient or population, generated from clinical data, that allows simulation, prediction, and testing of therapeutic interventions in silico [13,14]. This framework enables the creation of synthetic cohorts that mirror the demographic, clinical, and biological characteristics of real patients, thereby facilitating hypothesis testing and validation of treatment effects. Monte Carlo simulation provides a probabilistic method to model uncertainty by repeatedly sampling from distributions of input variables and calculating the likelihood of different outcomes [15,16]. When combined with a DT framework, it allows the estimation of variability, confidence intervals, and sensitivity to parameter changes across thousands of virtual iterations, enhancing the robustness and reproducibility of predictions.

We aimed to compare outcomes between DOAC and VKA therapy in USVT using real-world data, and to validate these findings through a DT-based Monte Carlo simulation framework, representing the first such application in this clinical context.

## 2. Materials and Methods

This observational, retrospective study was conducted in accordance with the Strengthening the Reporting of Observational Studies in Epidemiology (STROBE) declaration [17]. The protocol received approval from the Ethics Committee of Hospital Universitario Clínico San Carlos (code 25/277-O_M_SP). The study adhered to the Declaration of Helsinki (Fortaleza, Brazil, October 2013) and the Good Clinical Practice guidelines of the International Conference on Harmonization.

### 2.1. Patients and Study Setting

We included patients diagnosed with acute symptomatic USVT at Hospital Universitario Infanta Leonor (Madrid, Spain) between January 2012 and May 2024. Patients were identified through the electronic registries of the venous thromboembolism unit. Eligible patients were adults (≥18 years) with an objectively confirmed diagnosis of USVT. Patients with catheter-related thrombosis or incomplete follow-up data were excluded.

Basilic, cephalic, axillary, or subclavian vein thrombosis was diagnosed using Doppler ultrasonography (Vivid S70N, GE Healthcare, Chicago, IL, USA) or computed tomography (CT) (SOMATOM Definition AS, Siemens Healthineers, Erlangen, Germany). Portal, mesenteric, or splenic vein thrombosis was confirmed by ultrasonography, CT, or magnetic resonance imaging (MRI) (Achieva 1.5T, Philips Healthcare, Best, The Netherlands). CVT, involving the cerebral venous sinuses or veins, was confirmed by CT angiography or MRI.

The choice of anticoagulant—DOAC or VKA—was made by the treating physician according to clinical judgment. All patients receiving DOACs were prescribed full-dose regimens consistent with approved indications. For VKA therapy, the target INR was maintained between 2.0 and 3.0. No treatment switches occurred between groups during follow-up. Patients were followed for at least 12 months or until death, whichever occurred first. Data quality was routinely verified.

No formal sample size calculation was performed due to the study’s retrospective design. However, we included all USVT cases diagnosed during the study period, ensuring maximal case capture. Furthermore, the use of DT modeling allowed expansion of the synthetic cohort and Monte Carlo resampling, partially compensating for the limited number of real-world observations.

Missing data were handled using a complete-case approach. No imputation methods were applied, and each analysis used all available observations for the variables involved.

### 2.2. Baseline Variables

Baseline variables included demographic characteristics (age, sex), type of anticoagulant, and type of USVT, comorbidities (obesity, current smoker, hypertension, diabetes mellitus, dyslipidemia, chronic cardiovascular or pulmonary disease, prior stroke, estrogen therapy, chronic kidney disease, malignancy, and chronic liver disease) thrombophilia (antiphospholipid syndrome, factor V Leiden mutation, prothrombin G20210A mutation, protein C or S deficiency, and antithrombin deficiency), paroxysmal nocturnal hemoglobinuria, chronic myeloproliferative syndromes (polycythemia vera, essential thrombocythemia), and analytic variables measured at the time of USVT diagnosis (platelet count, hemoglobin level, and D-dimer). Additional variables included were time in therapeutic range [TTR] for patients receiving VKAs (calculated using the Rosendaal method), concomitant antiplatelet therapy, duration of follow-up, and vital status at the end of follow-up.

### 2.3. Outcomes

The primary outcome was the thrombotic recanalization rate, assessed by follow-up imaging performed within six months after diagnosis or earlier when available. Recanalization was defined according to the radiologist’s report, indicating complete or incomplete resolution of the initial thrombus. Imaging studies were reviewed by staff radiologists as part of routine clinical care, and assessors were not blinded to treatment allocation. Follow-up imaging included Doppler ultrasonography, CT, or magnetic resonance imaging (MRI), depending on the thrombosis site.

Secondary outcomes included thrombotic recurrence, defined as a new thrombosis in previously unaffected vascular territories confirmed by imaging, all-cause mortality, and major bleeding events. Major bleeding was defined following the International Society on Thrombosis and Haemostasis (ISTH) criteria as any overt hemorrhage requiring transfusion of at least two units of blood, occurring in retroperitoneal, spinal, intracranial, intrathecal, intrapericardial, or intraocular locations, or leading to death [18].

### 2.4. Digital Twin Generation and Validation

**Figure 1:**
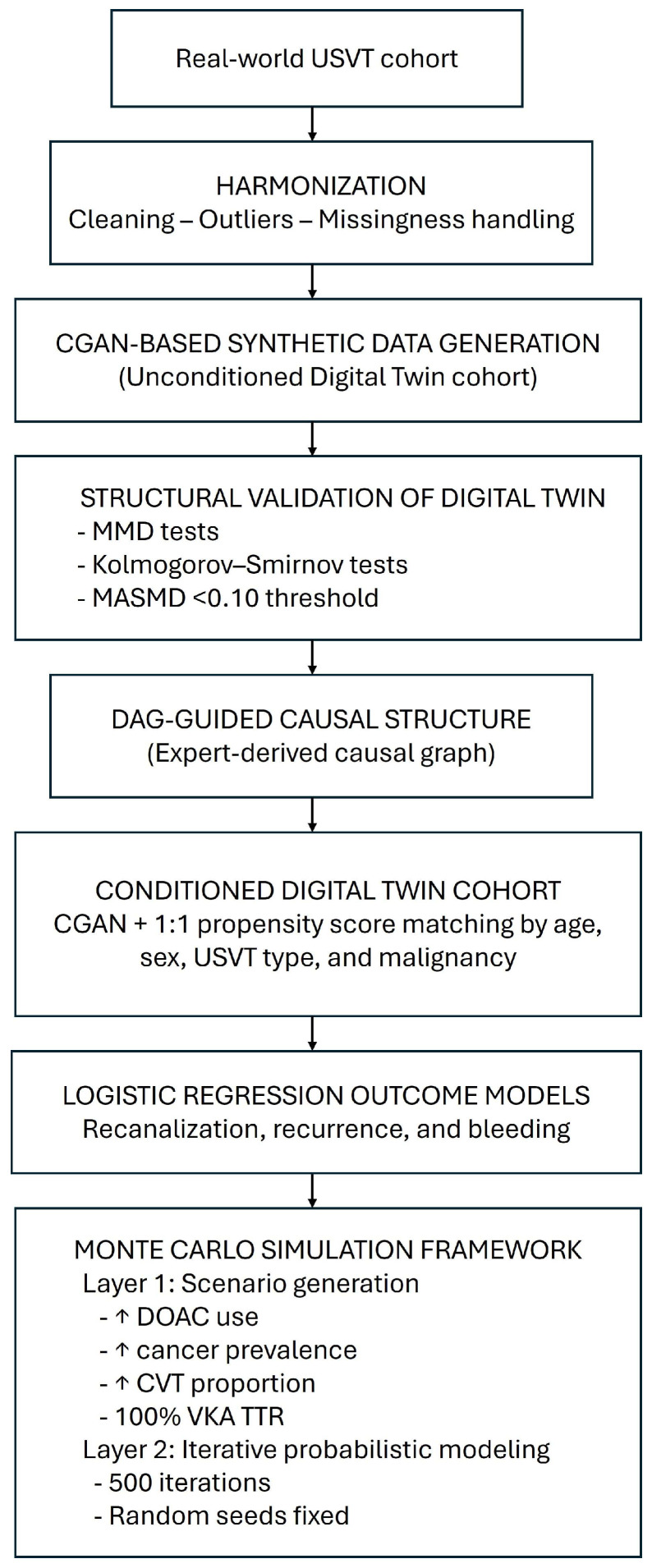
Overview of the digital twin generation workflow and Monte Carlo simulation framework. “↑” means that the percentage for that scenario increased.

Figure 1 shows the full DT generation pipeline, including data harmonization, CGAN-based synthesis, structural validation, DAG-guided causal modeling, conditional cohort creation, and integration with the Monte Carlo simulation framework.

#### 2.4.1. Synthetic Cohort Generation

DTs were generated using generative adversarial networks (GANs) and conditional GANs (CGANs) to emulate individual patient clinical profiles with USVT. The CGAN model generated a synthetic cohort of the same size as the original dataset (1:1 ratio), thereby preserving the original dataset’s population characteristics. Before model training, patient-level information was standardized to ensure uniform variable definitions, appropriate handling of missing data, and outlier identification. Synthetic data generation was conditioned on key clinical covariates, including USVT location, anticoagulant strategy (VKA or DOAC), age, sex, and malignancy status.

To evaluate fidelity, the DT cohort was compared with the original dataset by examining baseline variable distributions and observed clinical outcomes. The objective of this validation process was to confirm that the synthetic population retained both statistical coherence and clinical plausibility relative to the real-world USVT cohort. Maximum mean discrepancy (MMD) and the Kolmogorov-Smirnov test were applied to assess differences in the marginal distributions of core continuous and categorical variables between real and synthetic data.

#### 2.4.2. Structural Validation and Causal Coherence

A directed acyclic graph (DAG) was incorporated into the CGAN training framework to represent clinically relevant dependencies among variables and to minimize spurious associations. The DAG specified the assumed causal links between selected covariates and informed the structure of dependencies used during synthetic data generation. Variables included in the DAG were USVT type, treatment group, age, sex, malignancy, and outcomes (recanalization rate, thrombosis recurrence, and major bleeding). The DAG was constructed through expert consensus involving three clinicians and one data scientist, based on established causal relationships described in the thrombosis literature. No external dataset was available for independent DAG validation.

Outcomes were included in the DAG as terminal nodes to guide causal structure, but were not used as conditioning variables during CGAN training. The number of nodes and directed edges in the DAG was deliberately limited to maintain interpretability and prevent overfitting, in line with recommendations from causal inference frameworks emphasizing parsimony and clinical relevance [19]. The directionality of the edges was defined based on established evidence and clinical reasoning.

Embedding the DAG within the CGAN framework improved the clinical plausibility of the resulting DTs by explicitly encoding variable-to-variable dependencies, thereby enabling more robust and interpretable outcome simulations across the real-world USVT population.

#### 2.4.3. Conditional Cohort Creation and Model Evaluation

Following the generation of DTs, synthetic cohorts were created to simulate distinct treatment scenarios, enabling a comparison between DOAC- and VKA-treated patients under equivalent baseline conditions. Logistic regression was used to evaluate the association between anticoagulation type and outcomes, as well as to assess the internal consistency of the synthetic population.

Covariate balance between the real and synthetic cohorts was evaluated using mean absolute standardized mean differences (MASMDs), with a MASMD of less than 0.1 indicating adequate balance. Convergence was defined by stabilization of the generator–discriminator loss across epochs. An MASMD ≤ 0.20 was predefined as acceptable, while <0.10 was used as the target threshold. To explore the internal structure of the data, Spearman correlation matrices were computed for key covariates, enabling a comparison of dependency patterns between the original and synthetic cohorts.

The final validated synthetic dataset was used for outcome modeling and internal consistency assessment. DT generation required approximately 20–25 min per cohort on a workstation with GPU acceleration. Random seeds were fixed across Python v3.12, NumPy v2.0, and TensorFlow v2.16 for reproducibility, and each digital-twin replication used a distinct but controlled seed.

### 2.5. Outcome Modeling

Logistic regression models were used to estimate outcome probabilities for complete recanalization, thrombotic recurrence, and major bleeding across treatment scenarios in both real and synthetic cohorts.

Model calibration was verified by comparing predicted and observed outcome frequencies using Brier scores and calibration plots. Regression coefficients were inspected to ensure clinical interpretability and internal consistency with known predictors of outcomes.

### 2.6. Monte Carlo Simulation Framework

We implemented a Monte Carlo framework to quantify the uncertainty of outcome estimates derived from the conditioned DT cohort and to evaluate prespecified “what-if” clinical scenarios. The framework took as input the conditioned DT cohort and propagated uncertainty by repeatedly generating synthetic case-mixes, computing predicted outcome probabilities from the fitted models, and aggregating marginal risks over iterations.

#### 2.6.1. Scenario Generation (Layer 1)

Starting from the conditioned DT cohort, we created perturbed case-mixes by modifying the joint distribution of key predictors used in the outcome models (treatment group, site of index USVT, and active cancer status) while preserving their empirical dependencies via stratified resampling from the DT data (with fixed random seeds). Four scenarios were evaluated in addition to the baseline DT case-mix: (a) 70% DOACs/30% VKAs (inverse of the real-world distribution); (b) Cancer + 50% relative increase at baseline; (c) CVT 40% of all USVT cases; (d) 100% TTR for VKAs (applied by reassigning VKA patients to a perfect-control stratum for the risk models).

#### 2.6.2. Outcome Simulation (Layer 2)

For each iteration and scenario, binary logistic regression models fitted in the conditioned DT cohort were used to obtain predicted probabilities for the three outcomes: complete recanalization, thrombotic recurrence, and major bleeding. Complete recanalization was modeled as a binary endpoint (complete vs. not complete), using the distribution learned from patients with available follow-up imaging and applying it to the corresponding scenario case-mix. For each patient profile in the resampled cohort, a Bernoulli draw with the model—based probability generated the simulated outcome; marginal risks were then averaged across the cohort. Each scenario was run with R = 500 independent iterations. We report mean event rates with 95% confidence intervals computed from the empirical distribution across iterations. Random seeds were fixed to ensure reproducibility.

#### 2.6.3. Validation and Sensitivity Analyses

We monitored numerical stability using Monte Carlo standard errors (MCSEs) and coefficients of variation, targeting MCSE < 5% for all endpoints. Sensitivity analyses varied (i) the number of iterations and (ii) the resampling scheme for scenario generation; results were compared to baseline DT outputs to assess robustness. No time-to-event or competing-risk models were applied within the Monte Carlo engine; all simulations used the logistic-model—based probabilities defined above.

### 2.7. Statistical Analysis in the Original Cohort

Continuous variables were described using mean ± standard deviation (SD) or median and interquartile range (IQR), depending on distribution, as evaluated by the Shapiro-Wilk test. For comparisons between treatment groups (DOACs vs. VKAs), normally distributed continuous variables were assessed with Student’s *t*-test. In contrast, non-normally distributed variables were compared using the Mann-Whitney U test. Categorical variables were reported as counts and percentages and were analyzed using the chi-square test or Fisher’s exact test when expected cell frequencies were below five. Statistical significance was set at *p* < 0.05. Analyses were performed using SPSS Statistics version 29.0 (IBM Corp., Armonk, NY, USA) and R version 4.3.2 (R Foundation for Statistical Computing, Vienna, Austria; www.r-project.org, accessed on 1 July 2025). The same analytical framework was applied to the synthetic and simulated cohorts where applicable.

## 3. Results

### 3.1. Characteristics and Outcomes in Real-World Patients

Ninety patients with USVT were included: 65 (72.2%) received VKAs, and 25 (27.8%) received DOACs. Among those on DOACs, seven received apixaban, seven received edoxaban, six received rivaroxaban, and five received dabigatran. The distribution of USVT types was as follows: 55 patients (61.1%) had SVT, 33 (36.7%) UEDVT, and 2 (2.2%) CVT. In patients with SVT, PVT was the most frequent (47 cases), followed by mesenteric and splenic vein thrombosis (4 cases each).

Baseline characteristics are shown in Table 1. Forty-nine patients (54.4%) were female, with a mean age of 67.5 years (±17.7). The median duration of follow-up was 29.5 (interquartile range [IQR]: 20.1–43.7) months. The median time in TTR for VKA patients was 60.9% (IQR 51.4–70.8). The prevalence of hypertension, chronic liver disease, and active cancer was significantly higher in the VKAs group. SVT was more frequent in patients receiving VKAs (69.2% versus 40.0%), while UEDVT and CVT were more frequent among those receiving DOACs (52.0% versus 30.8%, and 8.0% versus 0%, respectively).

Follow-up imaging was available for 40 patients. 9 of 25 patients (36.0%) treated with VKAs achieved complete recanalization, compared with 6 of 15 patients (40.0%) receiving DOACs (*p* = 0.827). Within the DOAC type, complete recanalization was observed in 3 of 5 patients (60.0%) on dabigatran, 2 of 4 (50.0%) on apixaban, 1 of 2 (50.0%) on rivaroxaban, and none of 4 on edoxaban. During follow-up, symptomatic thrombotic recurrence occurred in 7.7% among those receiving VKAs (5 of 65) and 8.0% among those treated with DOACs (2 of 25) (*p* = 0.961). In the DOACs group, one recurrence occurred with dabigatran, and one with edoxaban. During follow-up, seven events (10.8%) of major bleeding in patients receiving VKAs group were observed, and two (8.0%) among those treated with DOACs (*p* = 0.695). Overall, 44 of 90 patients (48.9%) died during follow-up. Mortality was 39/65 patients (60%) treated with VKAs compared with 5/25 (20%) receiving DOACs (*p* < 0.001). Among deceased patients, 21 deaths in the VKA group and 2 in the DOAC group were cancer-related. Two fatal bleeding events occurred in patients treated with VKAs (one intracerebral hemorrhage and one massive hemoptysis), and one fatal gastrointestinal bleeding was reported in a patient receiving a DOAC. No patients died due to thrombotic recurrence.

In a stratified sensitivity analysis excluding patients with active cancer, mortality was substantially lower in both treatment groups. Among non-cancer patients, mortality was 25% in the VKA group (10/40) and 19% in the DOAC group (4/21).

### 3.2. Digital Twin

#### 3.2.1. Non-Conditioned Digital Twin Cohort and Internal Structure Validation

An initial non-conditioned DT cohort was generated using the real-world USVT dataset to evaluate the model’s internal structure and fidelity. Ten independent DT replicas were created to assess reproducibility. The distributions of key variables—age, sex, type of USVT, treatment group, and active cancer—closely matched those of the real cohort.

Across all synthetic cohorts, the absolute standardized mean differences (ASMDs) for individual variables were <0.1, and the overall mean ASMD (MASMD) was 0.069 (±0.038). In the internal comparison between DOAC and VKA subgroups within the DT, baseline covariates also demonstrated adequate balance (ASMD values: 0.061 for age, 0.052 for sex, 0.073 for USVT type, 0.086 for treatment group, and 0.057 for active cancer), with an overall MASMD of 0.057 (±0.025).

**Figure 2:**
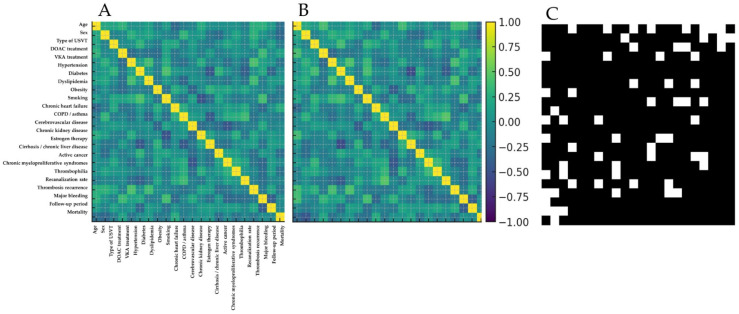
Spearman correlation matrices of encoded clinical variables: (**A**) real-world cohort; (**B**) CGAN-generated digital twin cohort; (**C**) pairwise correlations differing by >0.1 (white), illustrating high structural similarity.

Among all pairwise comparisons, 84.7% of correlations in the synthetic cohort differed by less than 0.1 in absolute value relative to the real data (Figure 2).

#### 3.2.2. Conditioned Twins and Treatment Effect Estimation

A conditioned DT cohort was constructed using CGAN-generated data combined with 1:1 propensity score matching based on age, sex, USVT type, and malignancy. After matching, covariate balance between the DOAC and VKA subgroups was satisfactory, with ASMDs of 0.026 for age, 0.028 for sex, 0.071 for USVT type, and 0.059 for active cancer. The overall MASMD was 0.054 (±0.028), confirming good balance within the conditioned synthetic cohort. The matched DT cohort had a mean age of 64.7 (±14) years, with 42% women. The incidence of malignancy was 34.4%. Logistic regression was applied to estimate the recanalization rate, thrombosis recurrence, and major bleeding under each treatment scenario. The model predicted a thrombotic recurrence rate of 8.6% for patients receiving VKAs and 10.9% for those treated with DOACs, resulting in an absolute difference of 2.3% in favor of VKAs. The complete recanalization during follow-up was 38.0% with VKAs and 40.3% with DOACs, a difference of 2.3% favor of DOACs. Major bleeding was 9.1% with VKAs and 7.6% with DOACs, an absolute difference of 1.5% in favor of DOACs. Across these iterations, MASMD values compared to the original dataset ranged from 0.048 to 0.102 (mean 0.069 ± 0.019), supporting the internal stability of the generative process and consistency of the treatment effect estimates.

#### 3.2.3. Sensitivity and Uncertainty Analysis

Outcome rates for each treatment group in the synthetic cohort were estimated. In the real-world cohort, recanalization data were available in 40 patients with follow-up imaging. In the DT models, this variable was extrapolated to the whole synthetic cohort using the distribution derived from these patients. The predicted complete recanalization during follow-up was 38.0% among patients receiving VKAs and 40.3% among those treated with DOACs, corresponding to an absolute difference of 2.3% in favor of DOACs (Table 2). The estimated thrombotic recurrence rate was 8.6% with VKAs and 10.9% with DOACs, an absolute difference of 2.3% in favor of VKAs. Major bleeding occurred in 9.1% of VKA-treated and 7.6% of DOAC-treated patients (1.5% in favor of DOACs). Overall, the predicted treatment effects were consistent with those observed in the real-world cohort, with no reversal of risk direction across endpoints. Variability in model outputs across the ten independent DT replications remained low, with predicted outcome rates differing by less than ±1.5%, supporting the internal stability and reproducibility of the conditioned DT model.

### 3.3. Monte Carlo Simulation Results

**Figure 3:**
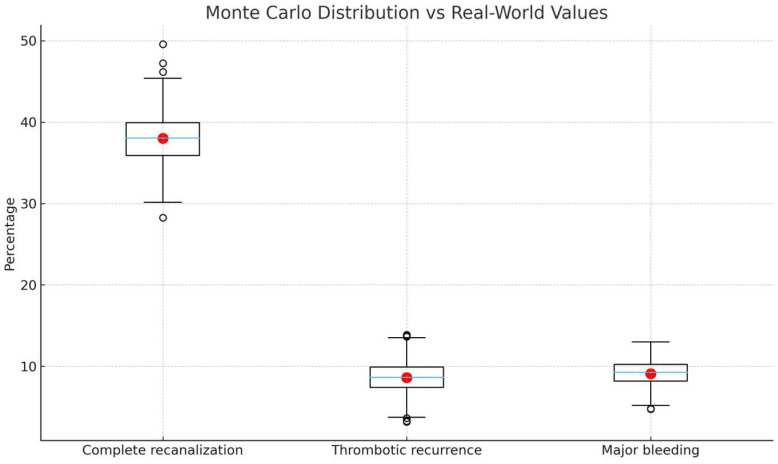
Monte Carlo output distributions (500 simulations) with real-world point estimates overlaid. Boxplots show simulated variability; red dots indicate observed cohort values.

Using the conditioned DT cohort as input, we performed R = 500 Monte Carlo iterations under four perturbation scenarios in addition to the baseline case-mix: (a) 70% DOACs and 30% VKAs (inverse of the real-world distribution); (b) +50% relative increase in the prevalence of active malignancy (from 34.4% to ~51.6%); (c) Increased proportion of CVT to 40% of all USVT cases; (d) 100% TTR for VKAs (Table 3). In each iteration, marginal outcome risks for each treatment group were recalculated using the logistic regression models. Mean outcome rates and 95% confidence intervals were obtained from the empirical distribution of 500 runs. All endpoints achieved MCSE < 5% and coefficients of variation < 10%, confirming numerical convergence and stability. Convergence was defined as stabilization of outcome variance below 5% across iterations, together with coefficients of variation < 10%, which was consistently achieved across all simulated endpoints. Detailed simulation summary statistics, including mean effect sizes, standard deviations, and 95% confidence intervals across 500 iterations, are provided in Supplementary Table S2. Directionality preservation was high: in 95% of the 500 Monte Carlo iterations, the estimated treatment effect retained the same direction as in the real-world cohort. A visual comparison between the Monte Carlo output distributions and real-world point estimates is provided in Figure 3.

Under Scenario (a), outcome rates were nearly identical to the baseline. In Scenario (b), thrombotic recurrence increased in both treatment groups, though the absolute difference between DOACs and VKAs remained stable (+2.2 percentage points). Scenario (c) (40% CVT prevalence) produced the highest event rates overall, particularly for recurrence (14.4% with DOACs vs. 12.1% with VKAs). Finally, in Scenario (d), improved anticoagulation control with VKAs reduced both recurrence (6.9%) and bleeding (8.0%), narrowing differences between treatments and nearly eliminating the recanalization advantage of DOACs (+0.3 percentage points).

## 4. Discussion

To our knowledge, this is the first study applying a DT strategy combined with Monte Carlo modeling to the field of thrombosis, specifically addressing outcomes in USVT. We employed this integrative approach to assess the comparative effectiveness of DOACs versus VKAs in this clinical setting.

This study provides a novel perspective on anticoagulation in USVT by integrating DT and Monte Carlo simulation into a unified analytical framework. Through this approach, we aimed to reproduce real-world data behavior and simulate outcomes, including recanalization rate, thrombosis recurrence, and bleeding risk under DOACs or VKAs.

In the real-world cohort, we found low rates of complete recanalization among patients with USVT treated with either DOACs or VKAs. In addition, the rates of recurrent thrombosis and major bleeding were comparable between the two anticoagulant strategies. These findings suggest comparable safety and efficacy profiles between treatments. Our results are consistent with other series, supporting the hypothesis that DOACs offer similar efficacy and safety to VKAs across different USVT subtypes [20,21,22]. Remarkably, the proportion of patients with cancer in the DOACs group was lower compared to the VKAs arm, reflecting the administrative restrictions that, for many years, limited the use of these anticoagulants for treating venous thrombosis in oncologic patients in Spain. Such a limitation may have influenced treatment allocation, explaining the underrepresentation of cancer patients among those receiving DOACs, despite current international recommendations that favor these agents over vitamin K antagonists in most cases of cancer-associated thrombosis.

Beyond case-mix differences, additional factors, such as more predictable pharmacokinetics, fewer drug-drug interactions, and greater adherence to DOAC therapy, may also contribute to the observed mortality gap. However, these mechanisms remain speculative and cannot be inferred causally in the absence of randomized allocation.

After stratifying for cancer status, the mortality difference was attenuated but not abolished, supporting the likelihood of residual confounding and indicating that the mortality imbalance should not be interpreted as a direct treatment effect.

USVT poses a therapeutic challenge due to the scarcity of robust evidence, as patients with CVT, SVT, and UEDVT are underrepresented in pivotal DOAC trials. Consequently, current treatment recommendations are frequently extrapolated from guidelines developed for venous thromboembolism. The evidence in USVT is fragmented, and guideline recommendations remain inconsistent, especially in patients with advanced chronic hepatic dysfunction [11,12,23].

A DT is a computational model designed to create a dynamic virtual representation of real-world patients, enabling the simulation of clinical trajectories and therapeutic responses under varying conditions. In the field of thrombosis, this approach allows for the replication of complex biological and clinical interactions using real-world data, offering an alternative framework for hypothesis testing in rare disorders. A recently published study applied this strategy to patients with antiphospholipid syndrome [24], demonstrating its potential to model treatment effects and outcome variability in rare thrombotic diseases. In our study, we developed a DT model to reproduce the clinical behavior of patients with USVT treated with DOACs and VKAs, providing a virtual environment to compare outcomes and assess recurrence risk. The DT cohort successfully replicated the outcome patterns of the real population, showing comparable rates of recanalization, recurrent thrombosis, and major bleeding, and confirming the internal consistency and predictive validity of the synthetic model.

Nonetheless, DT approaches remain susceptible to overfitting, especially when trained on modest observational cohorts. Moreover, GAN-generated variables may be difficult to interpret, which could limit their translational applicability in clinical decision-making. Our model does not incorporate temporal or longitudinal imaging features, limiting its ability to simulate dynamic changes in thrombus evolution or recanalization.

In medicine, Monte Carlo simulation has been increasingly applied to predict disease evolution, estimate event risk, and assess therapeutic strategies under uncertainty. In the field of thrombosis, this approach has been used to reproduce thousands of virtual patient trajectories based on real-world probabilities of recurrence or bleeding between DOACs and VKAs across hypothetical scenarios, quantifying the dispersion of outcomes [25]. Another study applied Monte Carlo models to evaluate the impact of variability in adherence, dosage, or INR control on thromboembolic risk, highlighting its value in exploring clinical heterogeneity and real-world variability [26]. We further advanced this methodology by integrating a Monte Carlo simulation layer, which enabled repeated probabilistic modeling of treatment scenarios and reinforced the robustness of the digital twin framework for predictive and exploratory analyses. Through this simulation, we tested four hypothetical scenarios: a progressive increase in DOAC use, a higher prevalence of cancer-associated thrombosis, a predominance of CVT cases, and optimal therapeutic range control among patients on VKAs. Across all simulated conditions, the outcome patterns remained consistent with those observed in the real cohort, confirming similar efficacy between DOACs and VKAs, a stable safety profile, and improved recanalization and bleeding outcomes associated with DOAC exposure. These results further validate the stability and predictive reliability of the combined digital twin and Monte Carlo modeling approach.

Future extensions include the development of dynamic digital twins that update iteratively as new clinical information becomes available, as well as integration of longitudinal imaging-derived features or Bayesian updating frameworks.

Compared with other machine-learning approaches—such as random forests or causal forests—DTs uniquely allow synthetic cohort expansion and scenario testing, whereas these models primarily focus on prediction rather than generative simulation

Follow-up imaging was available for less than half of the cohort, potentially biasing estimates of recanalization rates. Patients without imaging may differ clinically from those who underwent follow-up studies, and this incomplete ascertainment should be considered when interpreting outcomes.

Beyond USVT, DT frameworks hold significant potential to advance research on other rare thrombotic disorders, where evidence is equally limited. Conditions such as catheter-unrelated upper extremity thrombosis, renal vein thrombosis, ovarian vein thrombosis, Budd-Chiari syndrome, and catastrophic antiphospholipid syndrome share similar challenges, including low incidence, heterogeneous clinical presentations, and the impracticality of conducting adequately powered randomized trials. DT models could enable in silico evaluation of therapeutic strategies, facilitate risk stratification, and support comparative-effectiveness analyses when real-world cohorts are small or highly fragmented.

This study has several limitations. First, it was based on observational data with a modest sample size, particularly in the DOAC subgroup, which may limit the statistical power and the robustness of subgroup analyses. Nevertheless, the use of DT technology partially mitigates this limitation by enabling data harmonization, synthetic population expansion, and simulation of complex clinical scenarios. Second, the number of patients within each USVT subtype was insufficient to allow stratified analyses. Consequently, all subtypes were evaluated jointly, providing an overall rather than subtype-specific assessment of treatment effects. This heterogeneity is an inherent limitation and may diminish the biological interpretability of aggregated estimates. Nonetheless, the DT framework preserved the subtype distribution through conditional modeling and probabilistic resampling, thereby partially mitigating the impact of this limitation. Furthermore, the VKA group included a higher proportion of patients with active malignancy, partly reflecting historical reimbursement restrictions in Spain that limited DOAC use in cancer patients. This imbalance may have contributed to the observed mortality difference. Third, although the generative models preserved key multivariable relationships and showed good internal validity, synthetic data inevitably incorporate some degree of statistical noise. This may lead to minor discrepancies when compared with real-world estimates; however, such variability is intrinsic to DT modeling and prevents overfitting, thus enhancing generalizability. Fourth, the DAG embedded in the CGAN architecture was designed from clinical expertise rather than fully data-driven inference, which may introduce bias through potential misclassification or omission of relevant causal pathways. Fifth, several subtypes of USVT, such as renal vein or ovarian vein thrombosis, were not represented in the cohort. Therefore, caution is needed when generalizing the findings to all clinical contexts or to populations with distinct baseline risks. Finally, external validation of the digital twin model in independent multicenter cohorts is warranted to confirm its generalizability. Moreover, quality-of-life outcomes were not systematically assessed, precluding evaluation of patient-centered endpoints.

## 5. Conclusions

DT and Monte Carlo modeling approaches collectively provide an innovative and complementary framework. Their combined use may support individualized therapeutic decision-making in USVT. The DT strategy enabled individualized simulation of treatment responses and recurrence outcomes, showing high concordance with real-world observations and strong potential for precision medicine applications. Finally, the Monte Carlo simulations confirmed the probabilistic stability of DT-derived estimations across repeated resampling and uncertainty propagation. Together, these approaches outline a reproducible approach to explore therapeutic scenarios in rare prothrombotic disorders.

## Figures and Tables

**Table 1:** Clinical characteristics and outcomes of the real-world cohort of patients with unusual-site venous thrombosis.

Baseline Characteristics	Total (*n* = 90)	VKAs Group (*n* = 65)	DOACs Group (*n* = 25)	*p*-Value
*Demographic data*				
Age (mean ± SD)	67.5 (±17.7)	68.9 (±17.2)	63.9 (±18.6)	0.448
Female sex, *n* (%)	49 (54.4)	33 (50.8)	16 (64.0)	0.259
*Type of USVT, n (%)*				
SVT	55 (61.1)	45 (69.2)	10 (40.0)	0.016
UEDVT	33 (36.7)	20 (30.8)	13 (52.0)	0.087
CVT	2 (2.2)	0 (0)	2 (8.0)	0.075
*Previous conditions, n (%)*				
Hypertension	48 (53.3)	39 (60.0)	9 (36.0)	0.041
Diabetes	26 (28.9)	21 (32.3)	5 (20.0)	0.249
Dyslipidemia	40 (44.4)	29 (44.6)	11 (44.0)	0.958
Obesity (BMI ≥ 30 kg/m^2^)	18 (20.0)	10 (15.4)	8 (32.0)	0.078
Current smoker	31 (34.4)	23 (35.4)	8 (32.0)	0.810
Chronic heart failure	9 (10.0)	7 (10.8)	2 (8.0)	0.695
COPD/asthma	23 (25.6)	18 (27.7)	5 (20.0)	0.454
Cerebrovascular disease	11 (12.2)	8 (12.3)	3 (12.0)	0.986
Chronic kidney disease	13 (14.4)	8 (12.3)	5 (20.0)	0.352
Estrogen therapy	1 (1.1)	1 (1.5)	0	0.533
Cirrhosis/chronic liver disease	25 (27.8)	22 (33.8)	3 (12.0)	0.038
Active cancer	29 (32.2)	25 (38.5)	4 (16.0)	0.041
Paroxysmal nocturnal hemoglobinuria	0 (0)	0 (0)	0 (0)	—
Chronic myeloproliferative syndromes	1 (1.1)	0 (0)	1 (4.0)	0.279
*Laboratory parameters (median, IQR)* ^†^				
Hemoglobin (g/dL)	13.1 (11.8–14.4)	12.9 (11.6–14.2)	13.6 (12.3–14.9)	0.181
Platelets (×10^9^/L)	238 (182–296)	231 (178–282)	251 (190–315)	0.279
D-dimer (ng/mL)	2982 (2143–3921)	3123 (2256–4050)	2743 (2080–3615)	0.295
*Concomitant antiplatelet therapy*	7 (7.8)	6 (9.2)	1 (4.0)	0.407
*Any thrombophilia, n (%)*	19 (21.1)	14 (21.5)	5 (20.0)	0.873
Complete recanalization during follow-up, n (%) *	15/40 (37.5)	9/25 (36.0)	6/15 (40.0)	0.827
*Thrombosis recurrence during follow-up, n (%)*	7 (7.8)	5 (7.7)	2 (8.0)	0.961
*Major bleeding during follow-up, n (%)*	9 (10.0)	7 (10.8)	2 (8.0)	0.695
*Follow-up period (median, IQR)*	29.5 (20.1–43.7)	30.1 (20.3–45.2)	27.9 (19.8–42.1)	0.386
*Mortality, n (%)*	44 (48.9)	39 (60.0)	5 (20.0)	<0.001

^†^ At the time of thrombosis diagnosis. * Follow-up imaging was performed in 25 patients treated with VKAs and 15 treated with DOACs. Paroxysmal nocturnal hemoglobinuria testing was unavailable in approximately 64% of patients, diagnostic evaluation for chronic myeloproliferative syndromes was incomplete in 52% of the cohort, and a full thrombophilia panel was unavailable in 58% of cases. Abbreviations: BMI, Body Mass Index; COPD, chronic obstructive pulmonary disease; CVT, cerebral venous thrombosis; DOACs, direct oral anticoagulants; IQR, interquartile range; SD, standard deviation; SVT, splanchnic vein thrombosis; UEDVT, upper extremity deep vein thrombosis; USVT, unusual-site venous thrombosis; VKAs, vitamin K antagonists.

**Table 2:** Predicted outcomes in the conditioned digital twin cohort according to treatment type.

Outcome	VKAs, % (95% CI)	DOACs, % (95% CI)	Absolute Difference *
Complete recanalization	38.0 (30.8–45.6)	40.3 (33.5–47.3)	+2.3
Thrombotic recurrence	8.6 (4.8–13.6)	10.9 (6.7–16.5)	+2.3
Major bleeding	9.1 (5.1–14.5)	7.6 (4.1–12.8)	−1.5

* For recanalization and major bleeding, DOACs are favored; for thrombotic recurrence, VKAs are favored. Abbreviations: CI, confidence interval; DOAC, direct oral anticoagulant; VKA, vitamin K antagonist.

**Table 3:** Predicted outcome rates in Monte Carlo simulations under varying clinical scenarios.

Scenario	Outcome	VKAs %, 95% CI	DOACs %, 95% CI	Δ* (Percentage Points)
Baseline ^§^	Complete recanalization	38.1 [36.0–40.3]	40.4 [38.3–42.6]	+2.3
	Thrombotic recurrence	8.7 [7.9–9.6]	10.9 [10.0–11.9]	+2.2
	Major bleeding	9.1 [8.2–10.1]	7.6 [6.8–8.5]	−1.5
70% DOACs	Complete recanalization	38.0 [35.9–40.1]	40.2 [38.1–42.4]	+2.2
	Thrombotic recurrence	8.6 [7.7–9.5]	11.1 [10.1–12.2]	+2.5
	Major bleeding	9.2 [8.3–10.2]	7.6 [6.8–8.6]	−1.6
+50% Cancer	Complete recanalization	36.0 [34.0–38.2]	38.2 [36.1–40.4]	+2.2
	Thrombotic recurrence	10.8 [9.8–11.9]	13.0 [12.0–14.2]	+2.2
	Major bleeding	10.4 [9.4–11.5]	9.0 [8.1–10.0]	−1.4
40% CVT	Complete recanalization	31.5 [29.4–33.7]	33.8 [31.6–36.0]	+2.3
	Thrombotic recurrence	12.1 [11.0–13.2]	14.4 [13.2–15.6]	+2.3
	Major bleeding	11.7 [10.6–12.9]	10.2 [9.2–11.3]	−1.5
100% TTR for VKAs	Complete recanalization	40.0 [37.9–42.2]	40.3 [38.2–42.5]	+0.3
	Thrombotic recurrence	6.9 [6.1–7.8]	10.9 [10.0–11.9]	+4.0
	Major bleeding	8.0 [7.1–8.9]	7.6 [6.8–8.5]	−0.4

^§^ Digital twin conditioned cohort. Δ*: absolute difference in percentage points (DOAC versus VKA). For recanalization (beneficial outcome), Δ > 0 favors DOACs; for thrombotic recurrence and major bleeding (adverse outcomes), Δ < 0 favors DOACs. Abbreviations: CI, confidence interval; CVT, cerebral venous thrombosis; DOACs, direct oral anticoagulants; TTR, time in therapeutic range; VKAs, vitamin K antagonists.

## Data Availability

The original contributions presented in this study are included in the article/Supplementary Material. Further inquiries can be directed to the corresponding author.

## Supplementary Materials

The following supporting information can be downloaded at: https://www.mdpi.com/article/10.3390/clinpract15120237/s1, File S1: Supplementary Tables and Additional Methodological Details.

## Abbreviations

The following abbreviations are used in this manuscript:

CGAN	Conditional Generative Adversarial Network
CI	Confidence Interval
CT	Computed tomography
CVT	Cerebral Venous Thrombosis
DAG	Directed Acyclic Graph
DOAC	Direct Oral Anticoagulant
DT	Digital Twin
GAN	Generative Adversarial Network
INR	International Normalized Ratio
LMWH	Low-molecular-weight heparin
MRI	Magnetic resonance imaging
PVT	Portal Vein Thrombosis
RCT	Randomized controlled trial
SD	Standard Deviation
SVT	Splanchnic Vein Thrombosis
TTR	Time in Therapeutic Range
UEDVT	Upper Extremity Deep Vein Thrombosis
USVT	Unusual-Site Venous Thrombosis
VKA	Vitamin K Antagonist
