# Supplementary material for: A Digital Twin Strategy Combined with a Monte Carlo Simulation Framework to Predict Outcomes in Patients with Unusual-Site Venous Thrombosis Treated with Direct Oral Anticoagulants Versus Vitamin K Antagonists Using Data from Real-World Populations
Source: Clin Pract. 2025 Dec 17;15(12):237. doi: 10.3390/clinpract15120237 (PMC12732008; doi:10.3390/clinpract15120237)
Supplement: Supplementary file 1 [file clinpract-15-00237-s001.zip › clinpract-3967796-supplementary.pdf]

Supplementary Material for the Introduction Section

Randomized Controlled Trials Evaluating Direct Oral Anticoagulants in Unusual-Site Venous Thrombosis

Several randomized controlled trials (RCTs) have evaluated the efficacy and safety of DOACs for treating USVT [2–9] (Table S1). Among them, five focused on cerebral venous thrombosis (CVT). The RE-SPECT CVT trial was an international, multicentre, open-label, blinded-endpoint (PROBE) study on patients with CVT [2]. In this trial, 120 participants were randomly assigned in a 1:1 ratio to receive either warfarin or dabigatran. The target International Normalized Ratio (INR) for the warfarin group was between 2.0 and 3.0, whereas patients in the dabigatran arm received 150 mg twice daily for 6 months, following an initial lead-in period of 5 to 15 days of parenteral anticoagulation with either low-molecular-weight heparin (LMWH) or unfractionated heparin (UFH). There were no cases of recurrent CVT in either treatment group. However, one case of gastrointestinal bleeding in the dabigatran group (1.7% [95% confidence interval [CI], 0.0–8.9]) and two cases of intracerebral hemorrhage in the warfarin group (3.3% [95% CI, 0.4–11.5]) were reported. In a follow-up analysis, the complete recanalization was 44% for patients on dabigatran compared to 36% for those on warfarin. In comparison, partial recanalization rates were 42% and 49%, respectively, with no significant difference ($p = 0.44$). In the CHOICE-CVT trial, patients with CVT were randomly assigned to receive either dabigatran (44 patients) or warfarin (45 patients) after a 10–15-day period of parenteral anticoagulation [3]. By day 180, the dabigatran group recorded a higher incidence of recurrent thrombosis (18.2%) compared to the warfarin group (6.7%); however, these findings did not reach statistical significance ($p = 0.099$). Both groups experienced no major bleeding events, and recanalization rates were comparable (75% versus 82.9%, respectively). Another RCT enrolled 55 participants with CVT, who were randomly assigned to receive either rivaroxaban 20 mg once daily, warfarin (target INR 2.0–3.0), or LMWH for at least six months [4]. The primary composite outcome was symptomatic intracranial hemorrhage, major extracranial hemorrhage, or mortality. At six months, the rivaroxaban group experienced one intracerebral hemorrhage (3.8%) and two clinically relevant non-major extracranial bleeding events (7.7%), while no bleeding events or deaths occurred in the warfarin/LMWH group. No deaths were reported. The rivaroxaban group also had one case of recurrent CVT (3.8%), compared with none in the control group. Complete or partial recanalization was achieved in all patients in both groups. The study conducted by Maqsood et al. involved 45 CVT patients who were randomized to receive either rivaroxaban (20–30 mg daily) or warfarin for 3 to 12 months, after a brief initial course of parenteral anticoagulation [5]. There were no major bleeding events or recurrences of thrombosis in either group. By the six-month evaluation, recanalization was achieved in 86% of patients taking DOACs and 83% of those on warfarin. Finally, a subgroup analysis of the EINSTEIN-Jr phase 3 trial included 114 children with CVT who were randomized to receive either rivaroxaban or standard anticoagulation after initial heparin treatment [6]. None of the 73 rivaroxaban recipients and 1 (2.4%) of the 41 standard anticoagulant recipients had recurrent thrombosis after 3 months. Clinically relevant bleeding occurred in 5 (6.8%; all non-major and non-cerebral) rivaroxaban recipients and in 1 (2.5%; major [subdural] bleeding) standard anticoagulant recipient. Complete or partial sinus recanalization occurred in 18 (25%) and 39 (53%) in the rivaroxaban group and in 6 (15%) and 24 (59%) in the standard anticoagulant arm, respectively.

To date, one RCT has evaluated the role of DOACs for treating upper extremity deep vein thrombosis (UEDVT). In the ARM-DVT study, 52 patients with UEDVT received apixaban at 10 mg twice daily for 7 days, followed by 5 mg twice daily for 11 weeks [7]. The primary efficacy was the composite of a ninety-day rate of new or recurrent thrombosis and thrombosis-related

death. The primary safety outcome was the composite of major and clinically relevant non-major bleeding. The study concluded recruitment in November 2021; however, the results have not yet been provided.

Finally, two studies focused on patients with splanchnic vein thrombosis (SVT). The RIPORT trial evaluated rivaroxaban versus no anticoagulation in patients with chronic non-cirrhotic portal vein thrombosis (PVT) [8]. The study was terminated early at a median follow-up of 11.8 months after an interim analysis revealed a marked difference in thrombosis recurrence, reporting zero versus 19.7 events per 100 person-years in the rivaroxaban and control groups, respectively (p = 0.0008). Major bleeding occurred in two patients receiving rivaroxaban and one patient in the control arm. The RIVA-SVT100 was a prospective, single-arm, multicenter interventional study that assessed the safety and efficacy of rivaroxaban in 100 non-cirrhotic patients with acute SVT [9]. Participants received rivaroxaban 15 mg twice daily for 3 weeks, followed by 20 mg once daily for 3 months. At three months, complete recanalization was achieved in 47.3% of patients and partial recanalization in 36.3%, yielding an overall recanalization rate above 80%. The incidence of recurrent thrombosis and major bleeding was both 2.1%, and one death (1%) occurred, deemed unrelated to SVT.

Table S1. Randomized controlled trials evaluating direct oral anticoagulants in the treatment of venous thrombosis at unusual sites.

	RE-SPECT CVT [2]	CHOICE-CVT [3]	SECRET [4]	Maqsood et al. [5]	EINSTEIN-Jr CVT [6]	ARM-DVT* [7]	RIPORT [8]	RIVA-SVT100 [9]
USVT	CVT	CVT	CVT	CVT	CVT	UEDVT	Non-cirrhotic chronic PVT	Non-cirrhotic SVT (portal, mesenteric, and splenic veins)
DOAC	Dabigatran, 150 mg twice daily	Dabigatran, 150 mg twice daily	Rivaroxaban, 20 mg daily	Rivaroxaban, 20–30 mg daily	Rivaroxaban (bodyweight-adjusted dose)	Apixaban, 10 mg twice daily for 7 days, followed by apixaban, 5 mg twice daily	Rivaroxaban, 15 mg daily	Rivaroxaban, 15 mg twice daily for 3 weeks, followed by 20 mg once daily
Comparison	Warfarin	Warfarin	Warfarin or LMWH	Warfarin	VKA	Warfarin or LMWH	No anticoagulation	None (single-arm study)
Minimum period of anticoagulation, months	6	6	6	3	3	3	6	3
Sample size	120	89	55	45	114	357	111	100
Median follow-up, days	175	180	180	365	90	90	909	180
Recurrent thrombosis (DOAC/control group), n	0/0	8/3	1/0	0/0	0/1	NA	0/10	2
Intracranial hemorrhage (DOAC/control group), n	0/2	NA	1/0	0/0	0/1	NA	0/0	0
Major extracranial hemorrhage (DOAC/control group), n	1/0	0/0	2/0	0/0	0/0	NA	2/1	3
Conclusions	Dabigatran was non-inferior to warfarin	Dabigatran showed inferior efficacy compared with warfarin	Rivaroxaban was non-inferior to warfarin or LMWH	Rivaroxaban was non-inferior to warfarin	Rivaroxaban was non-inferior to VKA	—	Rivaroxaban was safe and effective compared with no anticoagulation in non-cirrhotic chronic portal vein thrombosis	—

Abbreviations: CVT, Cerebral venous thrombosis; DOAC, direct oral anticoagulant; LMWH, low-molecular-weight heparin; NA, not available; SVT, splanchnic vein thrombosis. UEDVT, upper extremity deep vein thrombosis; USVT, unusual-site venous thrombosis; VKA, vitamin K antagonist. *The study concluded recruitment in November 2021. However, the results are not yet published. Table adapted from Franco-Moreno et al., 2025 [16].

Supplementary Material for the Methods Section

Section 2.4.1 — Synthetic Cohort Generation

Training convergence was monitored through discriminator loss stabilization and visual inspection of sample distributions across epochs, ensuring the generation of stable synthetic samples over time.

Additionally, to prevent the generation of clinically implausible synthetic profiles, the digital twin model incorporated hard constraints and applied a post-generation plausibility filter, ensuring that no physiologically impossible combinations were present in the synthetic dataset.

Section 2.4.3 — Digital Twin Replication, Conditioning and Internal Validation

Continuous variables were winsorized at the 2.5th and 97.5th percentiles to minimize the influence of extreme outliers before model training and validation.

To ensure robustness and reproducibility, ten independent, non-conditioned digital twin cohorts were generated using the same CGAN configuration and training parameters.

All analyses involving digital twin generation and validation were conducted using Python version 3.9 (Python Software Foundation, Wilmington, DE, USA) and relevant statistical and machine learning libraries, including NumPy version 1.26.4 (NumPy Developers, USA), pandas version 2.2.2 (pandas Development Team, USA), scikit-learn version 1.5.2 (scikit-learn Developers, Paris, France), matplotlib version 3.9.2 (matplotlib Development Team, USA), and TensorFlow version 2.17.0 (Google LLC, Mountain View, CA, USA).

Table S2. Summary statistics from Monte Carlo simulations.

Outcome	Mean simulated effect (DOAC – VKA), %	SD of simulated effect	95% CI of simulated effect	Interpretation
Complete recanalization	+2.1	1.4	+1.0 to +4.6	DOACs slightly higher recanalization in most simulations
Thrombotic recurrence	+2.4	1.1	+0.3 to +4.7	Slightly higher recurrence under DOACs, consistent across majority of iterations
Major bleeding	−1.6	0.9	−3.2 to −0.1	DOACs show lower bleeding rates with narrow variance

Abbreviations: CI, Confidence Interval; DOAC, Direct oral anticoagulant; SD, Standard deviation; VKA, Vitamin K antagonist.
